# Response to content prevalence is not adolescent exposure in TikTok influencer food marketing surveillance

**DOI:** 10.1017/S1368980026102699

**Published:** 2026-05-20

**Authors:** Roxanne Dupuis, Aviva A. Musicus, Omni Cassidy, Marie A. Bragg

**Affiliations:** 1 Population Health, NYU Grossman School of Medicine, USA; 2 Nutrition, Harvard School of Public Health, USA; 3 Center for Science in the Public Interest, USA; 4 Marketing, NYU Stern School of Business, New York, USA

We thank the author for their interest in our work and for highlighting the importance of timely surveillance of food marketing on social media. We also agree with their central thesis that **content prevalence is not equivalent to adolescents’ exposure** and appreciate their suggested approach to bridge inference about adolescent exposure from content exposure data.

Their first suggestion, to weight prevalence estimates by views, is a straightforward approach to bridging this gap when we only have access to content prevalence data. However – and as they specify – ideally these would be reflective of adolescent engagement with the posts, which, given the type of data (i.e. publicly available social media content posted by highly followed influencers), may not be achievable.

Their second suggestion, to implement bounded sensitivity analyses for missing nutrient profiles, is particularly interesting given the amount of missing data for unbranded foods. This type of analysis could help set floor and ceiling values for the unbranded content, which, as the authors point out, could help better estimate the difference in the health profile between branded and unbranded food and beverages featured.

Their third suggestion, to audit posts for multiple products among a random subset, would provide quantitative evidence in support of the fact that by only selecting one product in each video, we underestimated the true extent of food content prevalence. Additionally, from a methodological perspective, conducting such an audit in a random subset of posts is practical given the time-intensive nature of social media content analysis, where coders must pay attention to the images shown, the audio and the written caption.

While these suggestions provide feasible approaches for approximating adolescent exposure from content prevalence data, more direct measurement of adolescent exposure is crucial. In fact, our collective work on food marketing and social media motivated a larger evaluation of the impact of state-level social media policies on exposure to food marketing among adolescents on social media. As part of this larger evaluation, adolescent participants have agreed to share their social media feeds (by screen-recording their time on social media), allowing for direct measurement of exposure to food content by adolescents^(1)^.

Additionally, we want to emphasise that while we agree that assessing adolescent exposure has important policy implications, this was outside the scope of our study. We want to reiterate that content prevalence data can in and of itself be beneficial to inform policy and regulation, by focusing the spotlight on those who produce social media content, rather than those (including adolescents) who consume that content. We need to hold social media companies, influencers and food brands accountable for the content posted online, especially given that the majority of American adolescents use TikTok and, in 2020, a third of TikTok users were estimated to be under the age of 14^(2,3)^. Given that our sample focused exclusively on the most popular TikTok influencers, the algorithm was likely showcasing their content. We previously reported that among the 89 influencers who posted food/beverage content, individual food/beverage posts, on average, had garnered > 9 million views and > 1 million ‘likes’ each^(4)^. Whether adolescents are the intended or actual audiences for these influencers^(5)^, influencers’ reach and persuasive power among adolescent viewers has been well-documented^(6,7)^, and these data can support evidence-based regulation.
